# Fabrication and Characterization of Planar-Type Top-Illuminated InP-Based Avalanche Photodetector on Conductive Substrate with Operating Speeds Exceeding 10 Gbps

**DOI:** 10.3390/s18092800

**Published:** 2018-08-25

**Authors:** Jheng-Jie Liu, Wen-Jeng Ho, Cho-Chun Chiang, Chi-Jen Teng, Chia-Chun Yu, Yen-Chu Li

**Affiliations:** 1 Department of Electro-Optical Engineering, National Taipei University of Technology, No. 1, Section 3, Zhongxial East Road, Taipei 10608, Taiwan; jjliu@mail.ntut.edu.tw (J.-J.L.); t105658037@ntut.edu.tw (C.-C.C.); 2 Tyntek Corp., No. 15, Kejung Rd., Chunan Science Park, Chunan, Miaoli County 350, Taiwan; dan@serv.tyntek.com.tw (C.-J.T.); ccyu@serv.tyntek.com.tw (C.-C.Y.); tommylee@serv.tyntek.com.tw (Y.-C.L.)

**Keywords:** avalanche photodetector (APD), separate absorption, grading, charge and multiplication (SAGCM), multiplication gain, modulation frequency, eye diagram

## Abstract

This paper presents a high-speed top-illuminated InP-based avalanche photodetector (APD) fabricated on conductive InP-wafer using planar processes. The proposed device was then evaluated in terms of DC and dynamic performance characteristics. The design is based on a separate absorption, grading, charge, and multiplication (SAGCM) epitaxial-structure. An electric field-profile of the SAGCM layers was derived from the epitaxial structure. The punch-through voltage of the SAGCM APD was controlled to within 16–17 V, whereas the breakdown voltage (V_BR_) was controlled to within 28–29 V. We obtained dark current of 2.99 nA, capacitance of 0.226 pF, and multiplication gain of 12, when the APD was biased at 0.9 V_BR_ at room temperature. The frequency-response was characterized by comparing the calculated 3-dB cut-off modulation-frequency (f_3-dB_) and f_3-dB_ values measured under various multiplication gains and modulated incident powers. The time-response of the APD was evaluated by deriving eye-diagrams at 0.9 V_BR_ using pseudorandom non-return to zero codes with a length of 2^31^-1 at 10–12.5 Gbps. There was a notable absence of intersymbol-interference, and the signals remained error-free at data-rates of up to 12.5 Gbps. The correlation between the rise-time and modulated-bandwidth demonstrate the suitability of the proposed SAGCM-APD chip for applications involving an optical-receiver at data-rates of >10 Gbps.

## 1. Introduction

Avalanche photodetectors (APDs) are essential components in optical fiber communication, featuring high sensitivity [1,2,3,4] based on internal current gain. Optical fiber communication systems are now expected to provide >10 Gb/s to meet the growing demand for bandwidth in local area networks [5,6,7], metropolitan areas, and long-haul optical links [8,9]. High-speed InP-based APDs are preferred over PIN-type photodetectors [10], particularly for conventional long-haul applications. The preferred solution in these situations is the separate absorption, grading, charge, and multiplication (SAGCM) structure, due to its low dark current [11,12,13,14], high quantum efficiency, and high gain-bandwidth product [15,16,17]. The importance of performance and reliability in these systems [18,19] has prompted research on epitaxy and device processing for the further development of high-performance and high-speed planar InP based SAGCM-APDs [20,21,22,23,24].

In this study, we developed a low-cost InP-based APD with SAGCM structure and calculated the electric field profile based on the characteristics of the epitaxial layers. A two-step zinc-diffusion was used to control the p-n profile and thickness of the multiplication layer. We used current–voltage measurements (with and without light illumination) and capacitance–voltage to characterize the DC performance of the proposed APDs. We compared the predicted cut-off 3-dB frequency (*f*_3-dB_) with measured values measured under various modulated incident power levels and multiplication gains. To confirm that this device meets OC-192 requirements, we obtained eye diagrams of the fabricated APD under 0.9 V_BR_ using pseudorandom non-return to zero (NRZ) code with a length of 2^31^-1 at bit rates of 10–12.5 Gbps. A correlation was observed between the rise time in the eye patterns and the modulated 3-dB bandwidth in the modulated frequency response. The DC and AC results demonstrate the suitability of the proposed SAGCM-APD chip in applications that involve optical receivers at data rates of >10 Gbps.

## 2. Experiments

### 2.1. APD Epitaxial Deposition and Characterization

**Figure 1:**
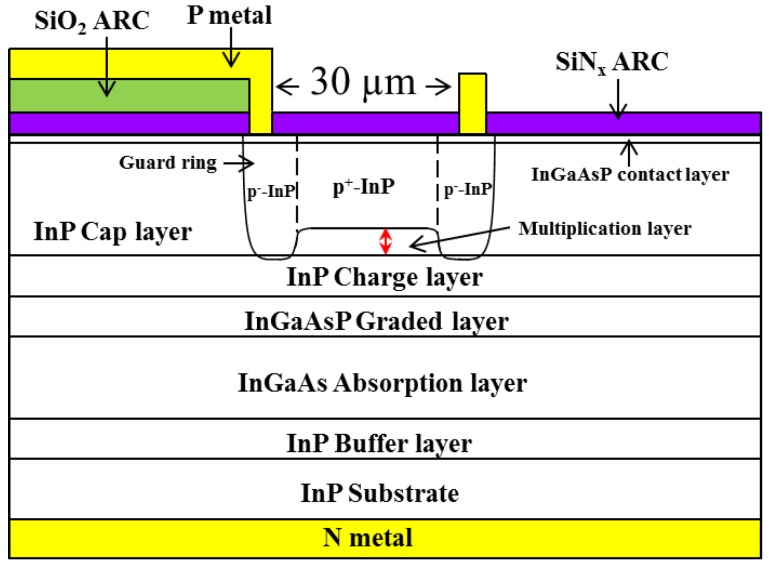
Schematic diagram showing the epitaxial layer structure of the proposed InP-based avalanche photodetector (APD) based on separate absorption, grading, charge, and multiplication (SAGCM).

Figure 1 presents a schematic diagram showing the separate absorption, grading, charge, and multiplication (SAGCM) epitaxial layer structure of the proposed InP-based APD. The epitaxial layers were grown on a (100)-oriented n^+^-InP substrate (S-doped) using metal organic chemical vapor deposition (MOCVD) under pressure of 100 mbar at a temperature of 600–650 °C. Arsine and phosphine were used as group-V source gases, whereas trimethyl-gallium and trimethyl-indium were used as group-III precursors. Disilane was used for n-type doping. The epitaxial layers of the proposed SAGCM-APD included a 1-μm-thick n-InP buffer layer (n ~ 5 × 10^17^ cm^−3^), a 1.2-μm-thick i-In_0.53_Ga_0.47_As absorbing layer (n < 5 × 10^15^ cm^−3^), three undoped InGaAsP grading layers (λ = 1.5, 1.3, 1.1 μm, 100 nm/each layer), a 0.15-μm-thick n-InP charge layer, and a 3.5-μm-thick undoped InP cap layer, and a 0.1-μm-thick undoped InGaAsP contact layer. The lattice mismatch between the InGaAs/InP and InGaAsP/InGaAs layers was less than 300 ppm (0.03%), as determined by double crystal X-ray diffraction (DXRD). Electrochemical capacitance–voltage (ECV) measurements indicate that the carrier concentration in the InP cap layer and middle In_0.53_Ga_0.47_As absorption layer were less than 5 × 10^15^ cm^−3^. The density of the InP charge layer was maintained at 3.5 × 10^12^ cm^−2^ [25]. The optical properties of the epitaxial layers were assessed using photoluminescence measurements at room temperature. The thickness measurements of the epitaxial layers and interface between the layers were confirmed using a scanning electronic microscope.

### 2.2. APD Device Fabrication and Characterization

Device fabrication began with the deposition of a 150 nm-thick SiNx layer on the surface of the APD epitaxial-structure substrate via plasma enhanced chemical vapor deposition (PECVD). A guard ring-window measuring 40 μm (mid-diameter) with 15-μm spacing was opened using reactive ion etching (RIE) to enable diffusion processing of the guard ring. A p^−^-InP guard ring was then formed using a Zn-diffusion process in a diffusion chamber at 530 °C for 5 min. After thorough cleaning, a new SiNx film was deposited on the surface of the sample, whereupon a central active window (35-μm diameter) covering the inner annulus of the guard ring was opened using RIE. A second Zn diffusion process was conducted at 570 °C for an extended duration to maintain the p^+^-InP diffusion front of the central active junction at a distance of 0.35 μm from the surface of n-InP charge layer, while simultaneously ensuring that the junction depth of the p^−^-InP guard ring under the SiNx reached the interface with the InP-cap/InP-charge layer using driven-in processes under constant source condition. After thorough cleaning, a λ/4-thick SiNx film was deposited on the surface of the APD device as an anti-reflective (AR) coating. A front-side p-electrode ring-metallization layer (AuZn (100 nm)/Ti (30 nm)/Au (300 nm)) was applied using photolithography, evaporation, and lift-off processing followed by rapid thermal annealing (RTA) at 425 °C for 60 s to ensure a good p-ohmic contact. In this work, evaporated Au-10 wt% Zn is not homogenous in depth, a two-layer structure with an Au layer lying above a Zn layer. The specific contact resistance of alloyed AuZn that underwent metallization to p-InGaAsP was approximately 1 × 10^−6^ Ω·cm^2^. Next, a metallization layer (Ti (30 nm)/Au (500 nm)) connecting the bond-pad to the p-electrode ring was deposited on the thick SiO_2_ layer (1–2 μm) to reduce parasitic capacitance. The sample was then thinned down to 150-μm to reduce series resistance. After back–side surface polishing and cleaning, AuGe films (100 nm)/Ni (30 nm)/Au (300 nm) were deposited on the n^+^-InP and subjected to annealing at 350 °C for 2 min to ensure a good n-ohmic contact. A schematic diagram showing the fabrication of the proposed APD is presented in Figure 1. Dark current–voltage (I-V), capacitance–voltage (C-V), and photo I-V measurements were used to characterize the DC performance of the APD. The time response and the frequency response were examined at the chip level (without packaging and with pre-amplification) using a microwave probe in conjunction with eye diagrams and 3-dB modulated frequency measurements.

### 2.3. Electric Field Profile Calculation

**Figure 2:**
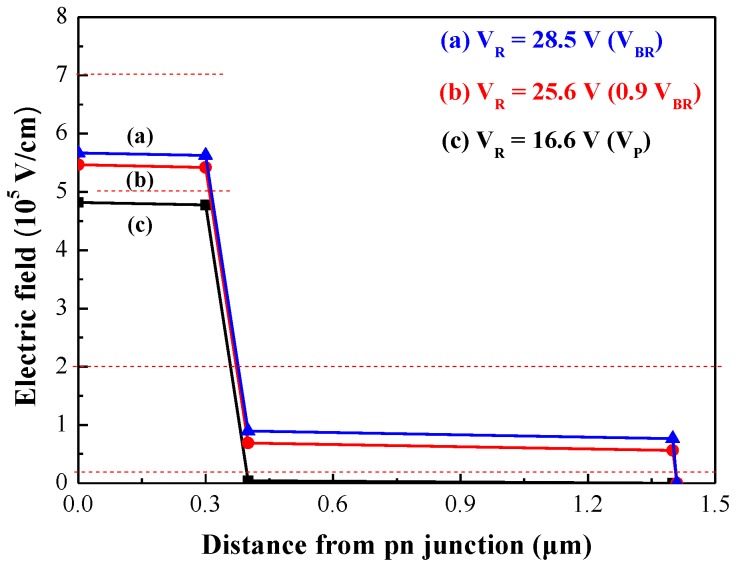
Electric field profile calculated as a function of the distance from the p-n junction under various reverse bias voltages.

In conventional InP-based SAGCM APDs, light absorption and carrier multiplication processes are kept separate by employing an absorption layer (InGaAs) with a small bandgap (E_g_ = 0.75 eV) and a multiplication layer (InP) with a large bandgap (E_g_ = 1.35 eV). Three InGaAsP grading layers are used to shift the bandgap from 0.75 eV to 1.35 eV to assist in the transport of carriers generated in the absorption layer into the multiplication layer. In an SAGCM structure, it is essential that the electric field distribution is optimized in the absorption and multiplication layers. The role of the charge layer is to maintain a high electric field for the multiplication layer and a low electric field for the absorption layer to prevent high-field induced current tunneling. Previous studies have reported maximum electric field intensities of 7 × 10^5^ V/cm in the n-InP multiplication layer and 2 × 10^5^ V/cm in the n^−^-InGaAs absorption layer [26], due to the generation of band-to-band current tunneling beyond this specific field intensity. Furthermore, the n-InP multiplication layer requires electric field intensity of >5 × 10^5^ V/cm to achieve carrier multiplication via impact ionization and rapidly sweep out the carriers generated in the n^−^-InGaAs absorption layer (i.e., before recombination). The electric field intensities in the n^−^-InGaAs absorption layer must exceed 2 × 10^4^ V/cm. Table 1 lists the structural parameters of the SAGCM-APD proposed in this work. Figure 2 presents the electric field profile, which was calculated as a function of the distance from p-n junction under various reverse bias voltages. The electric field intensity profile is well controlled to the required field range for the InP multiplication layer (5 × 10^5^–7 × 10^5^ V/cm) and the InGaAs absorption layer (2 × 10^4^–2 × 10^5^ V/cm).

## 3. Results and Discussion

**Figure 3:**
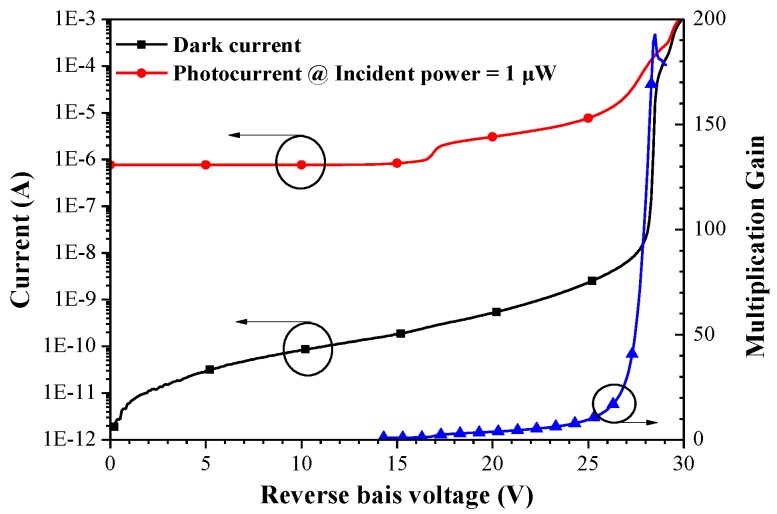
Dark current, photocurrent, and multiplication gain of conventional APD diodes as a function of reverse bias voltage.

Figure 3 presents the dark current, photocurrent, and multiplication gain of conventional APD diodes as a function of reverse bias voltage. The photocurrent was obtained under 1 μW illumination using a high-power 1550 nm distributed feedback laser diode (DFB-LD) source through an optical attenuator. The breakdown voltage (V_BR_) of 28.5 V is defined as the voltage under dark current (10 μA), whereas the punch-through voltage (V_P_) of 16.6 V is defined as the voltage applied to the APD with the depletion region extending into the InGaAs absorption layer at room temperature. The dark current (I_D_) was 2.99 nA at V_BR_ of 90% and 0.21 nA at V_P_. At a temperature of 300 K and illumination of 1 μW, we achieved responsivity of 9.61 A/W and multiplication gain of 11.9 at 0.9 V_BR_. In contrast, we obtained a responsivity of 23.30 A/W and multiplication gain of 28.1 at 0.95 V_BR_. The maximum multiplication gain at the V_BR_ was 192.

**Figure 4:**
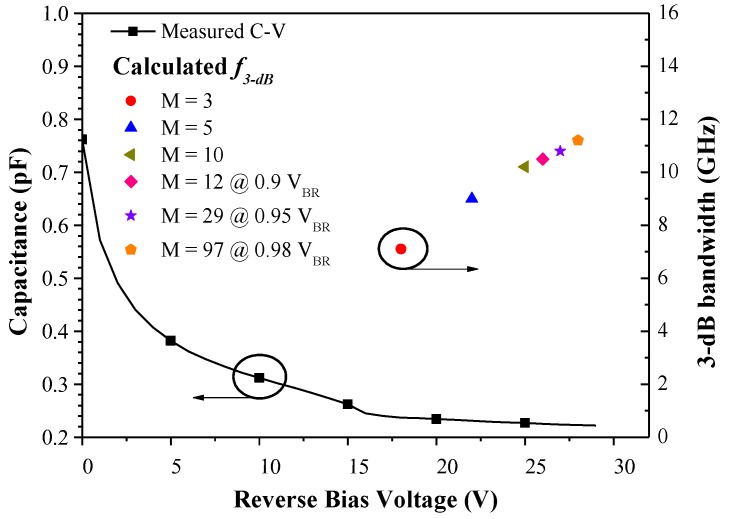
Capacitance and 3-dB frequency (*f*_3-dB_) calculated for the proposed APD (bonded on a ceramic sub-mount for testing) as a function of reverse bias voltage.

Figure 4 presents the capacitance and calculated 3-dB frequency (*f*_3-dB_) of the fabricated APD (bonded to a ceramic sub-mount for testing) as a function of reverse bias voltage. The total capacitance of the fabricated APD included the capacitance of the p-n junction (C_p-n_), the capacitance of the bond-pad (C_bp_), and the capacitance of the bridge-connection (C_bc_), in parallel connection mode. C_p-n_ depends on the area of the p-n junction and the applied reverse bias voltage. However, C_bp_ and C_bc_ depend on the area of the metallization electrode and the thickness of the dielectric film beneath the metallization electrode. The initial total capacitance under zero voltage was 0.726 pF, which decreased to 0.226 pF following the application of biasing at 0.9 V_BR_. We observed a kink in the capacitance–voltage (C-V) curves at 15–17 V, which is in good agreement with the V_P_ in the photo I-V curves under a punch–voltage of 16.6 V. We sought to predict the 3-dB bandwidth (*f*_3-dB_) frequency response of the proposed APD using C-V data, using the following equations:

(1) $$\frac{1}{{\left(f_{3-\mathrm{dB}}\right)}^{2}}=\frac{1}{{\left(f_{3{-\mathrm{dB}}_{RC}}\right)}^{2}}+\frac{1}{{\left(f_{3{-\mathrm{dB}}_{TR}}\right)}^{2}}$$

(2) $$f_{3{-\mathrm{dB}}_{RC}}=\frac{1}{2\pi RC}$$

(3) $$f_{3{-\mathrm{dB}}_{TR}}=\frac{0.45v}{L}$$

where *R* refers to the resistance of the load (50 Ω), *C* is the diode capacitance (depending on reverse bias voltage), *v* is the drift velocity in the InGaAs absorption layer, and *L* is the thickness of InGaAs absorption layer. *f*_3-dB_ was calculated as 7.09, 9.0, and 10.2 GHz at multiplication gains of 3, 5, and 10, respectively. *f*_3-dB_ was calculated as 10.5, 10.8, and 11.2 GHz under reverse bias voltages of 0.9, 0.95, and 0.98 V_BR_, respectively. Calculated *f*_3-dB_ values were compared with measured values to further examine avalanche build-up time.

**Figure 5:**
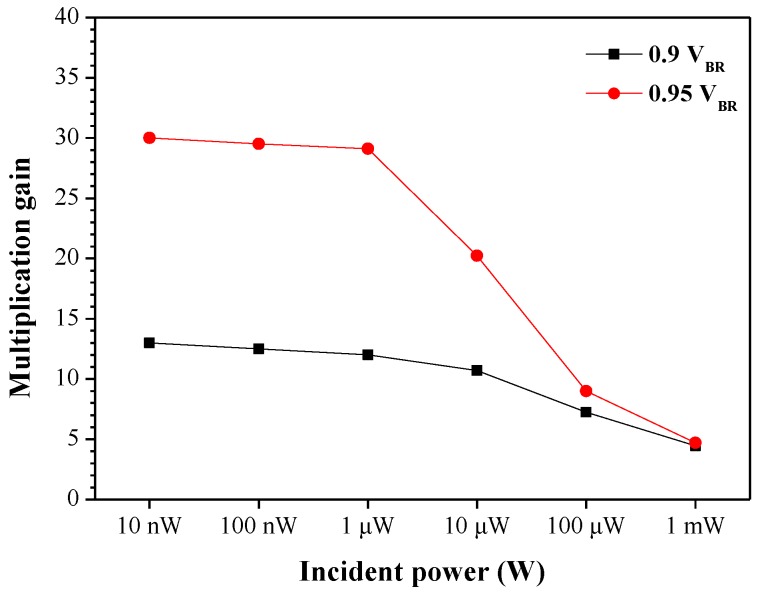
Multiplication gain as a function of incident power from 1 nW to 1 mW under bias voltages of 0.9 V_BR_ and 0.95 V_BR_.

Figure 5 presents multiplication gain as a function of incident power from 1 nW to 1 mW under bias voltages of 0.9 V_BR_ and 0.95 V_BR_. Multiplication gain was shown to decrease with an increase in incident power. APD biased at a higher voltage and illuminated using lower incident power resulted in far higher gain; however, we observed a significant decrease in multiplication gain when the incident power exceeded 1 μW. This is an indication that suitably low incident power was required for APD operations in regions of high multiplication gain.

**Figure 6:**
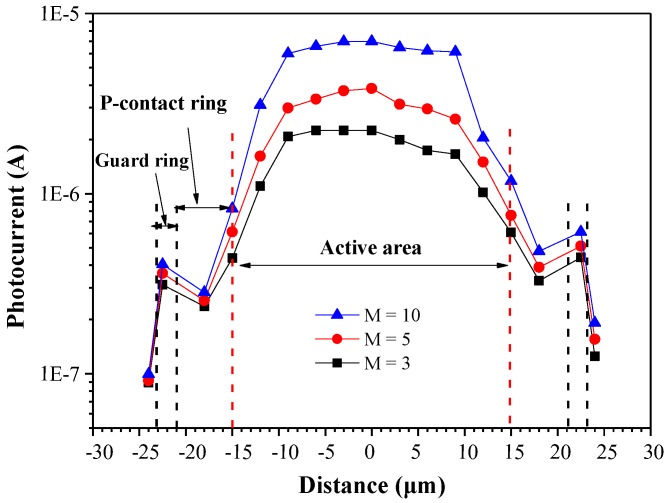
Photocurrent as a function of the distance that across the active area (the diameter) of the proposed SAGCM-APD.

Figure 6 presents the photocurrent as a function of distance across the active area (diameter) of the proposed SAGCM-APD. Incident light was delivered using a DFB laser via a fiber coupler lens at a wavelength of 1550 nm with power of 1 μW. The light-spot of the fiber coupler lens was moved in steps of 3 μm. Higher photocurrent values were obtained when the APD was operated using higher multiplication gain. We did not observe a significant spike at the edge of the active region in any of the photocurrent curves when the multiplication gain was varied between three and 10. This demonstrates the efficacy of the proposed structure in suppressing edge-breakdowns. Furthermore, the proposed APD achieved responsivity of 0.82 A/W when operating under multiplication gain of three to 10.

**Figure 7:**
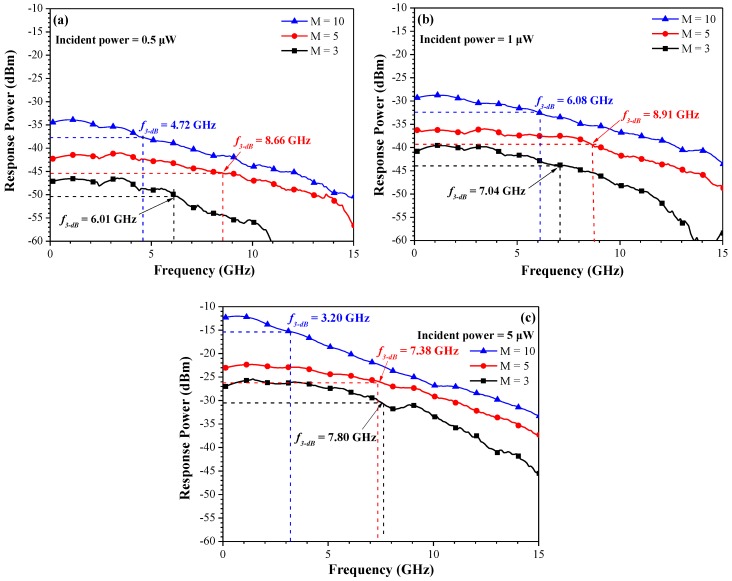
Measured *f*_3-dB_ values as a function of multiplication gain (3, 5, and 10) under incident optical powers of (**a**) 0.5 μ W, (**b**) 1.0 μW, and (**c**) 5.0 μW.

Figure 7 presents the measured *f*_3-dB_ as a function of multiplication gain (3, 5, and 10) under incident optical power of 0.5 μW, 1.0 μW, and 5.0 μW, as measured with load resistance of 50 Ω using an HP 8703A lightwave component analyzer. The response power of the APD increased with an increase in incident power. The response power of the APD also increased when the APD was operated under higher multiplication gain. The *f*_3-dB_ increased with an increase in multiplication gain from three to five and then decreased when the multiplication gain exceeded 10, in the cases where the incident power was 0.5 μW or 1.0 μW, as shown in Figure 7a,b. In contrast, *f*_3-dB_ decreased with an increase in the multiplication gain increased in the cases with an incident power of 5.0 μW, as shown in Figure 7c. In this work, we obtained a maximum *f*_3-dB_ of 8.91 GHz at an incident power of 1.0 μW and multiplication gain of five. At an applied incident power of 1.0 μW, the measured *f*_3-dB_ values when using multiplication gains of three and five were 7.04 and 8.91 GHz, respectively. In contrast, the calculated *f*_3-dB_ using the multiplication gains of three and five were 7.09 and 9.0 GHz, respectively. The measured *f*_3-dB_ values are in good agreement with the calculated *f*_3-dB_ values. In contrast, at an applied incident power of 1.0 μW and multiplication gain of 10, the measured *f*_3-dB_ was 6.08 GHz whereas the calculated *f*_3-dB_ was 10.2 GHz. The difference between 10.2 GHz and 6.08 GHz can be attributed to the considerable avalanche build-up time required under high multiplication gain.

**Figure 8:**
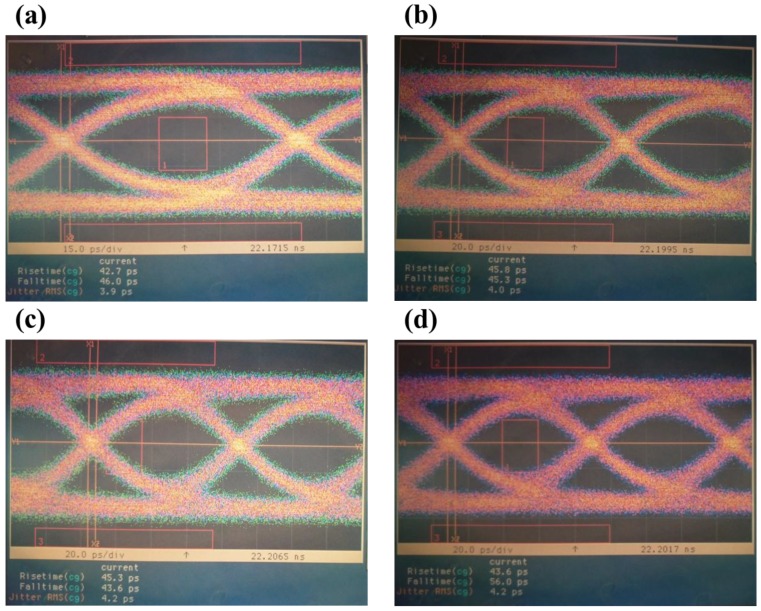
Eye diagrams obtained using the proposed SAGCM-APD photodetector chip operated under a multiplication gain of 5 using nonreturn-to-zero (NRZ) pseudorandom codes with length of 2^31^-1 at (**a**) 10, (**b**) 11, (**c**) 12, and (**d**) 12.5 Gb/s.

Applications that use an optical receiver with high bit rate require that devices be tested by superimposing a number of pseudorandom binary sequence (PRBS) patterns of ones and zeros. Figure 8 presents eye diagrams of the SAGCM-APD photodetector chip operated under a multiplication gain of five using nonreturn-to-zero (NRZ) pseudorandom codes with length of 2^31^-1 at (a) 10, (b) 11, (c) 12, and (c) 12.5 Gb/s. Note that the diagrams present the shape of an open human eye with the decision corresponding to the center of the opening. There was a notable absence of intersymbol interference and noise in the eye diagrams at bit rates of up to 12 Gb/s. As shown in Figure 8a, the rise time was 42.7 ps, the fall time was 46.0 ps, and the jitter was 3.9 ps when the APD was operated at a bit rate of 10 Gb/s. The overall quality of PRBS waveform patterns can be assessed simply by comparing an eye diagram against a predefined mask defining a set of keep-out regions into which the waveform must not intrude. As shown in Figure 8a, presenting the OC-192 mask in the eye diagram revealed that the waveform of the PRBS patterns did not intrude into the keep-out region. When the OC-192 mask was aligned to the 12 Gb/s eye diagram, only 0.1% of the waveform intruded into the keep-out region. This is a clear indication that the proposed SAGCM-APD is operable beyond 10 Gb/s and ideally suited to OC-192 optical fiber communication applications.

## 4. Conclusions

This paper outlines a 10 Gb/s planar-type top-illuminated InP-based avalanche photodetector with SAGCM-structure. High-quality epitaxial layers were deposited on a conductive InP substrate to form an SAGCM-structure. A two-step zinc-diffusion process was used to control the p-n profile and the thickness of the multiplication layer. Thick oxide was used to reduce bonding capacitance to enhance the frequency response. We obtained a maximum *f*_3-dB_ of 8.91 GHz at an incident power of 1.0 μW under multiplication gain of five. The proposed SAGCM-APD is ideally suited to OC-192 optical fiber communication applications at data rates exceeding 10 Gb/s. Our lab is currently involved in the development of a novel 25 Gb/s InP-based avalanche photodetector with SAGCM-structure based on the techniques demonstrated in the current study.

## Figures and Tables

**Table 1:** Structural parameters of separate absorption, grading, charge, and multiplication (SAGCM) avalanche photodetector (APD) proposed in this work.

Layer Name	Epitaxial Layer	Thickness (µm)	Concentration (cm^−3^)
Contact	n^−^-InGaAsP	0.1	undoped
Multiplication	n^−^-InP	0.35	undoped
Charge	n^+^-InP	0.15	<5 × 10^17^
Grading	n^−^-InGaAsP × 3	0.03 × 3	undoped
Absorption	n^−^-InGaAs	1.2	<5 × 10^15^
Buffer	n^+^-InP	1	5 × 10^17^
Substrate	n^+^-InP	350	5 × 10^18^

## References

[1] Ong D.S.G., Ng J.S., Hayat M.M., Sun P., David J.P.R. (2009). Optimization of InP APDs for high-speed lightwave systems. J. Lightw. Technol..

[2] Nakajima F., Nada M., Yoshimatsu T. (2016). High-speed avalanche photodiode and high-sensitivity receiver optical subassembly for 100-Gb/s ethernet. J. Lightw. Technol..

[3] Campbell J.C. (2016). Recent advances in avalanche photodiodes. J. Lightw. Technol..

[4] Ishimura E., Yagyu E. (2009). High sensitivity 2.5/10 Gbps InAlAs avalanche photodiodes. Mitsubishi Electr. Adv..

[5] Buckman L.A., Lemoff B.E., Schmit A.J., Tella R.P., Gong W. (2002). Demonstration of a small-form-factor WWDM transceiver module for 10-Gb/s local area networks. IEEE Photonics Technol. Lett..

[6] De Luces Fortes D.N., Pontes M.J., Giraldi M.T.M.R. Upgrading the transmission capacity of local area networks by improving the receiver performance. Proceedings of the SPIE.

[7] Fan S.-H., Chien H.-C., Chowdhury A., Chang G.-K. Spectrally efficient 60-GHz xy-MIMO data transport over a radio-over-fiber system for gigabit wireless local area networks. Proceedings of the 53rd IEEE Global Communications Conference.

[8] Poggiolini P., Bosco G., Benlachtar Y., Savory S.J., Bayvel P., Killey R.I., Prat J. (2008). Long-haul 10 Gbit/s linear and non-linear IMDD transmission over uncompensated standard fiber using a SQRT-metric MLSE receiver. Opt. Express.

[9] Das B., Abdullah M.F.L., Chowdhry B.S., Shah N.S.M. (2017). A Novel Signal regeneration technique for high speed DPSK communication systems. Wirel. Pers. Commun..

[10] Czuba K., Jurenczyk J., Kaniewski J. (2015). A study of InGaAs/InAlAs/InP avalanche photodiode. Solid-State Electron..

[11] Li B., Yang H.-W., Gui Q., Yang X.-H., Wang J., Wang X.-P., Liu S.-Q., Han Q. (2012). Ultralow dark current, high responsivity and thin multiplication region in InGaAs/InP avalanche photodiodes. Chin. Phys. Lett..

[12] Zhao Y., He S. (2012). The experimental investigation on dark current for InGaAs-InP avalanche photodiodes. Microelectron. Eng..

[13] Hwang S., Shim J., Yoo K. (2006). A 10-Gb/s planar InGaAs/lnP avalanche photodiode with a thin multiplication layer fabricated by using recess-etching and single-diffusion processes. J. Korean Phys. Soc..

[14] Kleinow P., Rutz F., Aidam R., Bronner W., Heussen H., Walther M. (2016). Charge-layer design considerations in SAGCM InGaAs/InAlAs avalanche photodiodes. Phys. Status Solidi A-Appl. Mat..

[15] Yoon K.H., Shin M.H., Park C.Y., Yun I., Kim S.J. (2004). Edge breakdown suppression of 10 Gbps avalanche photodiode. J. Korean Phys. Soc..

[16] Tarof L.E. (1990). Planar InP-InGaAs avalanche photodetectors with n-multiplication layer exhibiting a very high gain-bandwidth product. IEEE Photonics Technol. Lett..

[17] Rouvié A., Carpentier D., Lagay N., Décobert J., Pommereau F., Achouche M. (2008). High gain × bandwidth product over 140-GHz planar junction AlInAs avalanche photodiodes. IEEE Photonics Technol. Lett..

[18] Ishimura E., Yagyu E., Nakaji M., Ihara S., Yoshiara K., Aoyagi T., Tokuda Y., Ishikawa T. (2007). Degradation mode analysis on highly reliable guardring-free planar InAlAs avalanche photodiodes. J. Lightw. Technol..

[19] Yagyu E., Ishimura E., Nakaji M., Ihara S., Mikami Y., Itamoto H., Aoyagi T., Yoshiara K., Tokuda Y. (2009). Design and characteristics of guardring-free planar AlInAs avalanche photodiodes. J. Lightw. Technol..

[20] Burm J., Choi J.Y., Cho S.R., Kim M.D., Yang S.K., Baek J.M., Rhee D.Y., Jeon B.O., Kang H.Y., Jang D.H. (2004). Edge gain suppression of a planar-type InGaAs-InP avalanche photodiodes with thin multiplication layers for 10-Gb/s applications. IEEE Photonics Technol. Lett..

[21] Hyun K.-S., Paek Y., Kwon Y.-H., Hwang S., Shim J., Ahn S.J. (2004). Pre-breakdown suppression in planar InP/InGaAs avalanche photodiode using deep floating guard ring. Appl. Phys. Lett..

[22] Wei J., Dries J.C., Wang H., Lange M.L., Olsen G.H., Forrest S.R. (2002). Optimization of 10-Gb/s long-wavelength floating guard ring InGaAs-InP avalanche photodiodes. IEEE Photonics Technol. Lett..

[23] Vasileuski Y., Malyshev S., Chizh A. (2008). Design considerations for guardring-free planar InGaAs/InP avalanche photodiode. Opt. Quantum Electron..

[24] Yue A.-W., Wang R.-F., Xiong B., Shi J. (2013). Fabrication of a 10 Gb/s InGaAs/InP avalanche photodiode with an AlGaInAs/InP distributed bragg reflector. Chin. Phys. Lett..

[25] Kleinow P., Rutz F., Aidam R., Bronner W., Heussen H., Walther M. (2015). Experimental investigation of the charge-layer doping level in InGaAs/InAlAs avalanche photodiodes. Infrared Phys. Technol..

[26] Hyun K.S., Park C.Y. (1997). Breakdown characteristics in InP/InGaAs avalanche photodiode with p-i-n multiplication layer structure. J. Appl. Phys..

